# Effects of different ischemic preconditioning strategies on physiological and cellular mechanisms of intestinal ischemia/reperfusion injury: Implication from an isolated perfused rat small intestine model

**DOI:** 10.1371/journal.pone.0256957

**Published:** 2021-09-03

**Authors:** Yuk Lung Wong, Ingmar Lautenschläger, Lars Hummitzsch, Karina Zitta, François Cossais, Thilo Wedel, Rene Rusch, Rouven Berndt, Matthias Gruenewald, Norbert Weiler, Markus Steinfath, Martin Albrecht

**Affiliations:** 1 Department of Anesthesiology and Intensive Care Medicine, University Hospital Schleswig-Holstein, Kiel, Germany; 2 Institute of Clinical Chemistry, University Hospital Schleswig-Holstein, Kiel, Germany; 3 Institute of Anatomy, Christian-Albrechts-University, Kiel, Germany; 4 Department of Visceral and Thoracic Surgery, University Hospital Schleswig-Holstein, Kiel, Germany; University of Cambridge, UNITED KINGDOM

## Abstract

**Background:**

Intestinal ischemia/reperfusion (I/R)-injury often results in sepsis and organ failure and is of major importance in the clinic. A potential strategy to reduce I/R-injury is the application of ischemic preconditioning (IPC) during which repeated, brief episodes of I/R are applied. The aim of this study was to evaluate physiological and cellular effects of intestinal I/R-injury and to compare the influence of in-vivo IPC (iIPC) with ex-vivo IPC (eIPC), in which blood derived factors and nerval regulations are excluded.

**Methods:**

Using an established perfused rat intestine model, effects of iIPC and eIPC on physiological as well as cellular mechanisms of I/R-injury (60 min hypoxia, 30 min reperfusion) were investigated. iIPC was applied by three reversible occlusions of the mesenteric artery in-vivo for 5 min followed by 5 min of reperfusion before isolating the small intestine, eIPC was induced by stopping the vascular perfusion ex-vivo 3 times for 5 min followed by 5 min of reperfusion after isolation of the intestine. Study groups (each N = 8–9 animals) were: iIPC, eIPC, I/R (iIPC group), I/R (eIPC group), iIPC+I/R, eIPC+I/R, no intervention/control (iIPC group), no intervention/control (eIPC group). Tissue morphology/damage, metabolic functions, fluid shifts and barrier permeability were evaluated. Cellular mechanisms were investigated using signaling arrays.

**Results:**

I/R-injury decreased intestinal galactose uptake (iIPC group: p<0.001), increased vascular perfusion pressure (iIPC group: p<0.001; eIPC group: p<0.01) and attenuated venous flow (iIPC group: p<0.05) while lactate-to-pyruvate ratio (iIPC group, eIPC group: p<0.001), luminal flow (iIPC group: p<0.001; eIPC group: p<0.05), goblet cell ratio (iIPC group, eIPC group: p<0.001) and apoptosis (iIPC group, eIPC group: p<0.05) were all increased. Application of iIPC prior to I/R increased vascular galactose uptake (P<0.05) while eIPC had no significant impact on parameters of I/R-injury. On cellular level, I/R-injury resulted in a reduction of the phosphorylation of several MAPK signaling molecules. Application of iIPC prior to I/R increased phosphorylation of JNK2 and p38δ while eIPC enhanced CREB and GSK-3α/β phosphorylation.

**Conclusion:**

Intestinal I/R-injury is associated with major physiological and cellular changes. However, the overall influence of the two different IPC strategies on the acute phase of intestinal I/R-injury is rather limited.

## Introduction

Ischemia/reperfusion (I/R)-injury commonly results in organ failure and is associated with high mortality. An immediate and early recovery of blood flow (reperfusion) in affected areas is important to prevent irreversible organ damage. Paradoxically reperfusion in ischemic organs like heart, brain kidney and intestine increases the initial tissue damage leading to so called I/R-injury [1,2]. Especially in the intestine, I/R-injury caused by abdominal open and endovascular surgery, embolism and arteriosclerosis is often associated with a poor outcome for the affected patients [3]. Up to now the underlying cellular and molecular mechanism of intestinal I/R-injury are only partially understood and few studies investigated the intestinal pathophysiology during and after I/R-injury. However, intestinal I/R-injury often results in impaired epithelial/endothelial barrier function, reduced bowel movement and malabsorption leading to intestinal bacterial translocation with an increased risk for sepsis and organ failure [3,4]. Although some recent studies implicate ameliorated intestinal damage by remote ischemic preconditioning (RIPC) [5,6], no clinically established therapy options are available so far to reduce intestinal I/R-injury.

In case of the heart, brain, kidney and recently intestine, the application of ischemic preconditioning (IPC) and RIPC has the potential to reduce I/R mediated cell damage, morbidity and mortality in patients and animal models. Up to now, numerous animal studies and small clinical “proof-of-concept” trials have shown the benefits of different IPC strategies [5–10]. During IPC repeated, brief episodes of I/R are applied and protect the respective organ from a following fatal I/R-injury. On cellular level various complex organ dependent reactions are involved in the protective effects of IPC. On the final path IPC may activate ATP sensitive calcium channels and reduce mitochondrial calcium overload which plays a pivotal role in cell damage [11,12].

In this study we employed an isolated perfused rat small intestine model for a detailed analysis of the early physiological and cellular processes induced by intestinal I/R-injury. In addition, potential protective effects of IPC were evaluated using two different experimental approaches: (i) a physiological in-vivo IPC (iIPC) setup was used for protecting the intestine from I/R-injury. (ii) blood derived factors and nerval regulations were excluded by performing ex-vivo IPC (eIPC). We hypothesize that iIPC and eIPC differentially affect physiological as well as cellular events triggered by intestinal I/R-injury.

Our results show that although I/R-injury leads to major changes in intestinal physiology, effects of both strategies of IPC in the acute phase of intestinal I/R-injury are rather limited.

## Methods

### Animals

Female Wistar rats (180–220 g; Charles River, Sulzfeld, Germany) were used for all experiments. Animals were housed with standard diet and water ad libitum for at least 24 hours before surgery. The experiments were approved by the local authority (Ministry of Energy, Agriculture, Environment, Nature and Digitalization of the State of Schleswig-Holstein, Kiel, Germany; V242-65408/2015). All methods were conducted in accordance with the relevant guidelines and regulations. The recommendations of the ARRIVE guidelines (Animal Research: Reporting of In Vivo Experiments; https://arriveguidelines.org) were employed to improve the reporting of the study results. Organ isolation was performed under inhalation anaesthetics (1–6% Sevoflurane, for details see below) and intraperitoneal injection of Ketamine up to a maximum dose of 40 mg/kg. The rats remained narcotised until intestine extraction was performed. Euthanasia was elicited by cervical dislocation.

### Preparation of the small intestine, cannulation and perfusion

**Fig 1:**
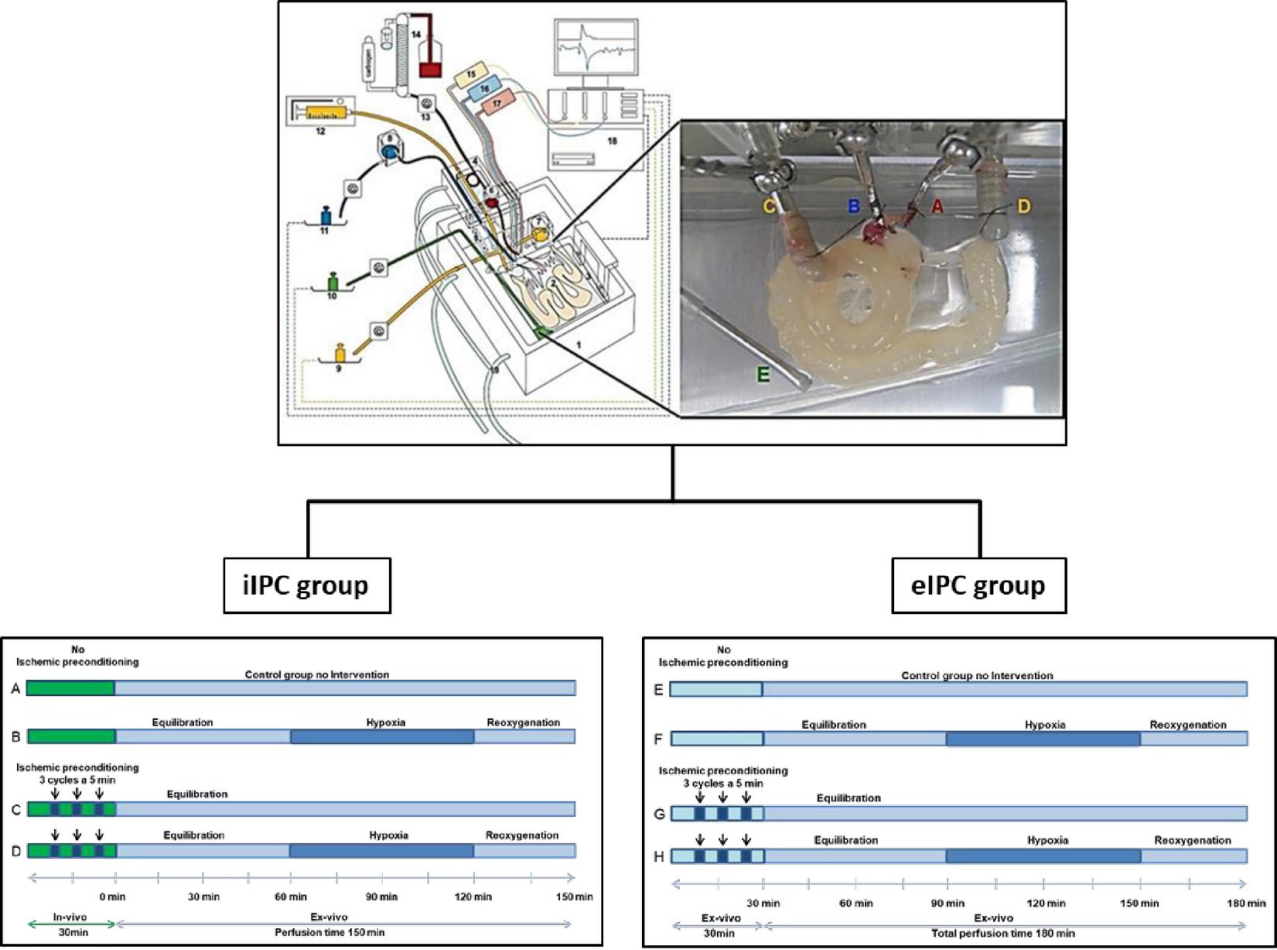
Experimental setting. Upper panel, schematic diagram of the isolated perfused intestine model. Using a custom made, heated chamber (1) an isolated small intestine (2) is perfused (vascular system: red; luminal system: yellow) while placed on a build-in microbalance (3). A moveable cannulating block (4) carries the tubings, heat exchanger cannula holders (5), and bubble trap (6). Height-adjustable reservoirs (7,8) allow clamping both afterloads to zero. For online analysis of fluid homeostasis all emanating liquids are quantified by use of three balances (9 to 11). Constant flow perfusion is applied by a syringe pump (12) and roller pump (13). The vascular perfusate is prewarmed, oxygenated, and pH equilibrated with tempered hollow fiber dialyzer flushed with carbogen gas (14). Pressure transducer (15–17) allow online detection of the luminal (yellow), venous (blue) and arterial (red) pressures. All data are recorded on a personal computer (18). To secure constant temperature, the chamber and cannulating block are water-jacketed and warmed by water bath (19). The inset shows a representative photograph of a perfused small intestine. A, arterial cannula; B, venous cannula; C, oral intestinal lumen cannula; D, aboral intestinal lumen cannula; E, lymphatic suction needle. Lower panel, experimental setup with treatment groups. In-vivo and ex-vivo ischemic conditioning were performed by inducing no flow ischemic conditions. In-vivo: Three reversible occlusions of the mesenteric artery using a vascular clamp for 5 min followed by 5 min of reperfusion before isolation of the small intestine. Ex-vivo: Interruption of the vascular perfusion by turning off the pumping system of the perfusion chamber for 5 min followed by 5 min of reperfusion repeated for three times. Ex-vivo ischemia/reperfusion damage was induced by replacing O_2_ in the perfusion buffer by N_2_/CO_2_ for a total time period of 60 minutes. Flow was kept constant during that period so that physiological parameters could be analyzed. Replacing O_2_ in the perfusion buffer by N_2_/CO_2_ results in a reduction of pO_2_ from initially >600mmHg to values <60mmHg. Since there are no oxygen carriers in the perfusion buffer, this value is equivalent to severe hypoxic conditions.

The detailed surgical procedure and ex-vivo perfusion was described by our group earlier [13,14]. Briefly, rats were anesthetized by inhalation Sevoflurane and additional intraperitoneal injection of Ketamine. In the filling phase of the anesthesia chamber, 6% Sevoflurane was initially administered and the animal was briefly (< 3min) transferred into the chamber. Further anesthetic management during surgery was conducted by using a rat anesthesia mask and maximum Sevoflurane concentrations of 1.0–1.5%. After opening the abdomen by a midline incision, further preparation steps were performed under a binocular microscope. Parts of the duodenum, the jejunum and ileum were isolated for the perfusion as described in detail for the rat [15]. All animals were killed under narcosis by cervical dislocation. The cannulation system used for perfusing the intestine was based on our recently published rat model [16]. Briefly, we used a custom-made perfusion system from Hugo Sachs Elektronik-Harvard Apparatus (March-Hugstetten, Germany) with modified cannulas and weight sensors. For perfusion, the aorta (in close proximity to the superior mesenteric artery), the hepatic portal vein as well as the proximal and distal small intestine were cannulated. For perfusion, the vascular perfusate was a modified Krebs-Henseleit solution containing 2 mM lactobionatic acid, 7.4 mM glucose, 30 mM mannitol, 0.8 mM glutamine, 122 μg/l norepinephrine hydrochloride, 12.6 mM HEPES, and 3% Albumin (BSA, Sigma-Aldrich, Munich, Germany) the osmolality was adjusted to 310–320 mosmol/l. The luminal perfusate contained 114 mM NaCl, 5 mM KCl, 26 mM NaHCO3, 30 mM lactose, 5.55 mM glucose, 10 mM mannitol, and 0.8 mM glutamine; the osmolality was adjusted to 310–320 mosmol/l. Perfusion of the small intestine was performed as described by Lautenschläger et al. [1] with minor modifications. Briefly, the single-pass perfusion via Tygon tubes and roller pump transported the vascular buffer for oxygenation through a dialyzer (FX paed, Fresenius Medical Care, Bad Homburg, Germany) with a rate of 2 ml/min. Constant luminal flow perfusion was applied by a syringe pump set to 0.06 ml/min. Vascular and luminal flow was continuously applied throughout the experiment without intermittent stops. The venous, luminal and lymphatic effluent weights were determined by high sensitive weight units connected to an online monitoring system (Hugo Sachs Elektronik-Harvard Apparatus, March-Hugstetten, Germany). A height adjustable reservoir for the venous and luminal outflow enabled sampling and reduction of afterload to prevent tissue damage and edema formation. The venous, lymphatic and luminal effluent volumes as well as the arterial, venous, and luminal perfusion pressures were recorded continuously. A blood gas analyser (Gem Premier 3000, Instrumentation Laboratory, Kirchheim, Germany) was used to measure O_2_ and CO_2_ partial pressures, pH, electrolytes, glucose and lactate of the arterial inflow and venous outflow every 15 minutes [10,11] (Fig 1).

### Experimental protocol of the in-vivo IPC (iIPC) group

The control group (A: control group, N = 8) received a vascular perfusion without intervention for 150 min. The second group (B: I/R group, N = 8) received after the equilibration phase of 60 min a hypoxic perfusion for 60 min followed by 30 min normoxic reperfusion (reoxygenation). In the third group (C: iIPC group, N = 8) iIPC was applied by three reversible occlusions of the mesenteric artery for 5 min followed by 5 min of reperfusion before isolation of the small intestine followed by 150 min of normoxic perfusion. The last group (D: iIPC+I/R group, N = 9) was treated with iIPC followed by an equilibration phase and I/R. All perfusions were continuously applied for 150 min without intermittent stops (Fig 1).

### Experimental protocol of the ex-vivo IPC (eIPC) group

In accordance to the iIPC group, the timeframes for eIPC, equilibration, hypoxia and normoxic reperfusion (reoxygenation) were matched (Fig 1). eIPC was performed by interrupting the vascular perfusion for 5 min followed by 5 min of reperfusion repeated for three times after isolation of the small intestine. The following eIPC groups were investigated: E: control group, N = 8. F: I/R group, N = 8. G: eIPC group, N = 8. H: eIPC+I/R group, N = 8 (Fig 1).

### Evaluation of fluid shifts, metabolic function and morphology

Details about the respective methods have been published recently [13,17]. Briefly, to determine the intestinal uptake of galactose, the luminal perfusion buffer was supplemented with 30 mM of lactose. Vascular galactose (derived from the luminal lactose) was determined by a commercially available assay kit (Raffinose/D-Galactose Assay Kit, Megazyme, Bray, Ireland). Vascular pyruvate was determined in the venous outflow by an enzymatic photometric method (NADH method, Sigma-Aldrich, Munich, Germany). For the measurement of vascular lactate a blood gas analyzer was employed (Gem Premier 3000, Instrumentation Laboratory, Kirchheim, Germany).To evaluate endothelial and epithelial permeability of the intestine, 40 mg/l FITC-dextran (150 kDa; Sigma-Aldrich, Munich, Germany) were added to the vascular perfusate. Samples of venous, lymphatic, and luminal outflow were collected every 15 min and analysed for the FITC-dextran content using a fluorescence ELISA reader (excitation 485 nm, emission 530 nm, Sunrise, Tecan, Crailsheim, Germany). For the determination of the intestinal wet weight, a 3 cm long proximal portion of the small intestine without mesentery and intestinal content was obtained before and after perfusion. The dry weight was determined after dehumidifying the samples for 96 h at 55°C. Additionally, at the end of perfusion a 3 cm long segment of the intestine was fixed in 4% formaldehyde for histological examination in which sections of intestinal tissue were stained with hematoxylin-eosin and periodic acid-Schiff [18]. Analyses of tissue damage were performed by a blinded investigator employing a histological stability score (histo-score) based on the evaluation of epithelial integrity [16]. Employing hematoxylin-eosin and periodic acid-Schiff stained samples, goblet cell ratios were calculated by analyzing 100 villi per specimen. The number of intestinal villi with villi tip coverage > 50% of goblet cell were divided by the number of villi < 50% of goblet cell coverage.

### Determination of apoptosis by Caspase-3/7 activity and Tunel staining

Activities of the effector Caspases-3 and -7, which play a central role in apoptotic events were evaluated in luminal effluent samples before and after perfusion using rhodamine based fluorometric assays (Apo-One homogeneous Caspase-3/7 assay, Promega Corporation, Madison, WI, USA). Treatment of the samples and evaluation of Caspase-3/7 activity were done based on the manufacturer’s protocol using a fluorescence ELISA reader (Tecan, Crailsheim, Austria) in combination with the Magellan software v1.1. Protein extraction was performed with RIPA buffer containing 150 mM sodium chloride, 1% NP-40, 1% sodium deoxycholate, 0.1% sodium dodecyl sulfate (SDS) and 50 mM Tris-HCl (pH 7.6; all from Sigma-Aldrich, Munich, Germany). Protein concentrations were determined with Roti®-Quant assays (Carl Roth, Karlsruhe, Germany) [13]. To visualize apoptotic cell nucleus in intestinal tissue sections, Tunel staining was performed by using a apoptosis detection kit (ApopTag Peroxidase In Situ, Merk, Darmstadt, Germany) and DAB substrate (Liquid DAB + Substrate Chromogen System, Dako, CA, USA) as described by the manufacturer.

### Proteome profiler array

For the evaluation of relative levels of phosphorylation of mitogen-activated protein kinases and other serine/threonine kinases phospho-MAPK arrays (ARY002B; R&D Systems; Minneapolis, MN, USA) were used. Samples of each respective group were pooled and analyzed based on the protocol provided by the manufacturer. Therefore, depending on the group, equal volumes of 8 or 9 samples were pooled (i.e. 50μl of each sample) and applied to the respective array membrane. Images were taken and the intensities of the signals were densitometrically analyzed with the software ImageJ (v1.48v, NIH). Signals on the membrane were compared with the corresponding reference spots and signals below 20% of the mean intensities of the reference spots were excluded from further analyses. Proteins on the array membranes are represented by duplicate spots and signal intensities shown in the results section represent the mean of the signal intensities of these two spots.

### Statistical analyses

All statistical analyses were performed using GraphPad Prism 5 for Windows (GraphPad Software, San Diego, USA). Data are presented as mean values with standard deviations (SD). Normal distribution was assessed by the Kolmogorov-Smirnov normality test. Statistical comparisons were performed using repeated measures two-way ANOVA with Bonferroni post-tests (galactose uptake, lactate-to-pyruvate ratio, vascular pressure, venous flow) as well as one-way ANOVA (parametric data) with Bonferroni post-tests or Kruskal-Wallis tests (non-parametric data) with Dunns post-tests (goblet cell ratio, Caspase 3/7 activities, luminal flow rate, luminal FITC-dextran transfer). Differences were considered to be statistically significant if p was less than 0.05. The number of animals used per experimental group was determined on the basis of experience and data from previous studies with the perfused intestine model, as well as a power analysis using the Graph Pad StatMate 2.00 program (GraphPad Software, San Diego, USA).

## Results

**Fig 2:**
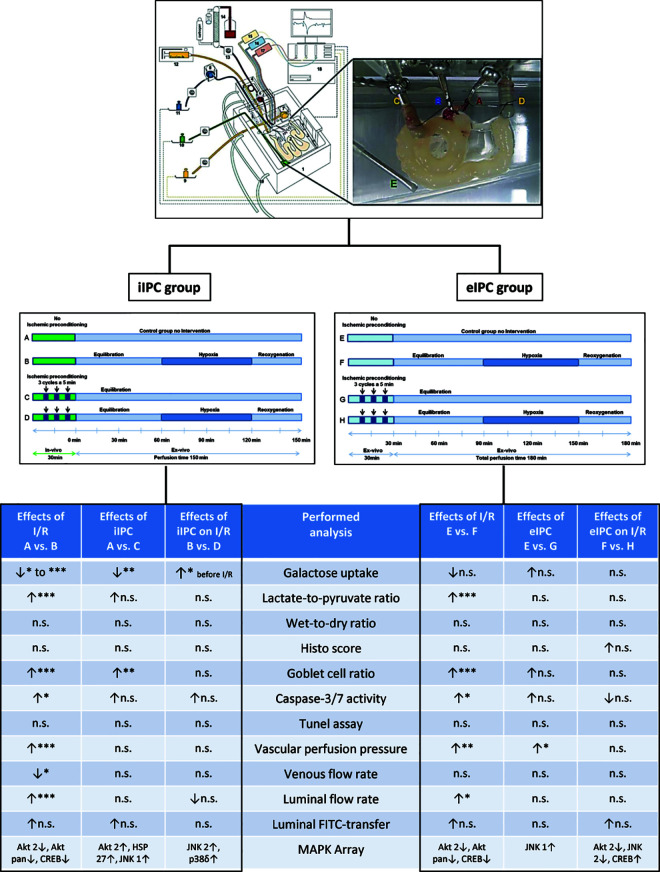
Experimental setting and summary of the results. n.s., not significant; *p<0.05; **p<0.01; ***p<0.001.

A summary of the main results of the study is shown in Fig 2. For details please refer to the respective subsections.

### Effects of I/R and IPC on intestinal galactose uptake

**Fig 3:**
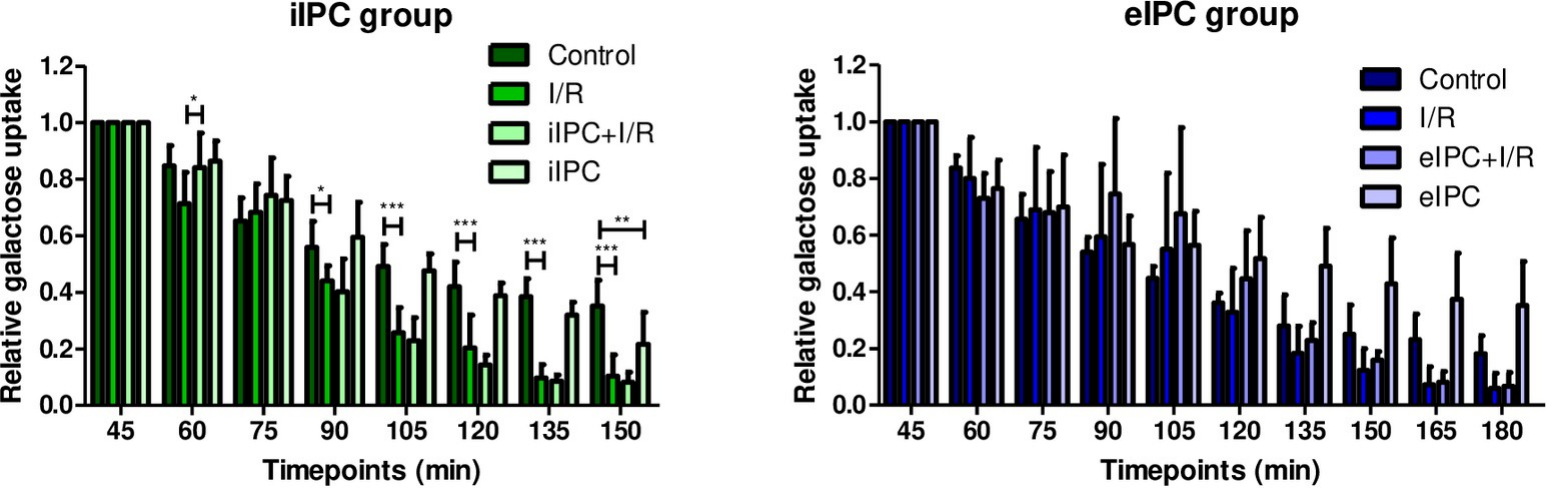
Relative galactose uptake in iIPC and eIPC groups. Bars denote the mean + SD. iIPC: Control (N = 8), I/R (N = 7), iIPC+I/R (N = 8), iIPC (N = 7); eIPC: Control (N = 5), I/R (N = 7), eIPC+I/R (N = 6), eIPC (N = 7). *p<0.05; **p<0.01; ***p<0.001.

Galactose uptake (derived from luminal lactose) as indicator for metabolic function showed a time dependent decrease in all groups which was possibly related to effects of the ex-vivo perfusion procedure on intestinal metabolism. I/R resulted in an additional decrease of galactose uptake which was most evident at later time points (iIPC: I/R_t90_, p<0.05; I/R_t105_, I/R_t120_, I/R_t135_, I/R_t150_ all p<0.001 vs. respective control). iIPC increased intestinal galactose uptake prior induction of I/R (iIPC+I/R_t60_, p<0.05), suggesting an enhanced intestinal functional performance at early but not at later time points, while eIPC had no effect at any of the time points investigated (Fig 3).

### Effects of I/R and IPC on lactate to pyruvate ratio

**Fig 4:**
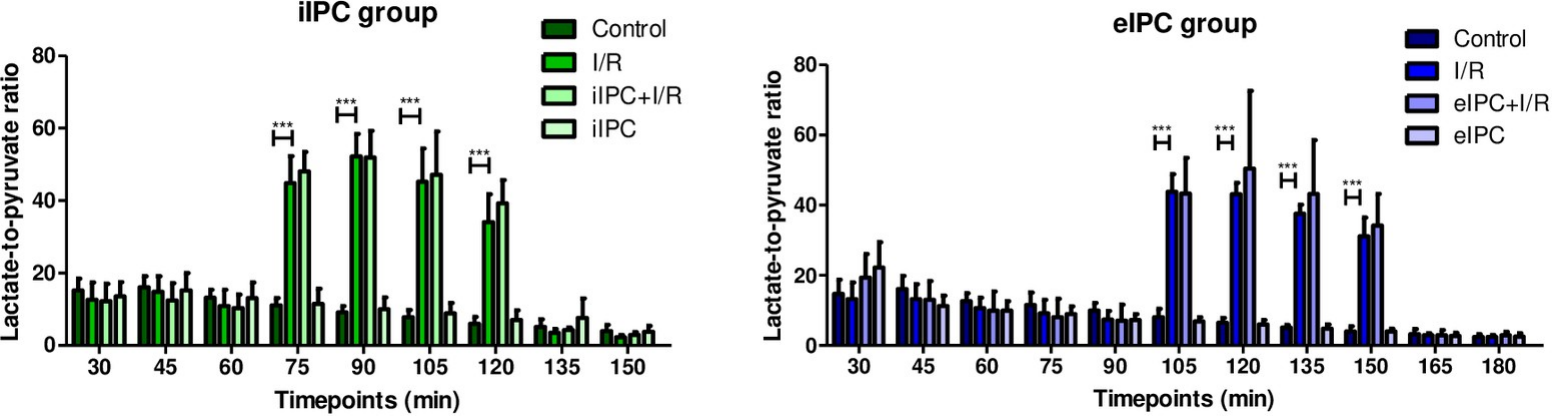
Lactate-to-pyruvate ratio in iIPC and eIPC groups. Bars denote the mean +SD. iIPC: Control (N = 7), I/R (N = 7), iIPC+I/R (N = 8), iIPC (N = 7); eIPC: Control (N = 5), I/R (N = 7), eIPC+I/R (N = 6), eIPC (N = 7). ***p<0.001.

The lactate-to-pyruvate ratio as parameter for intestinal function and aerobe/anaerobe metabolism showed a model related decrease during perfusion over the time in all groups. Significant changes in the time response signatures were present in the I/R groups during hypoxia and normalized quickly during reoxygenation (iIPC: I/R_t75_, I/R_t90_, I/R_t105_, I/R_t120_ all p<0.001 vs. respective control; eIPC: I/R_t105_, I/R_t120_, I/R_t135_, I/R_t150_ all p<0.001 vs. respective control; Fig 4).

### Effects of I/R and IPC on the number of goblet cells

**Fig 5:**
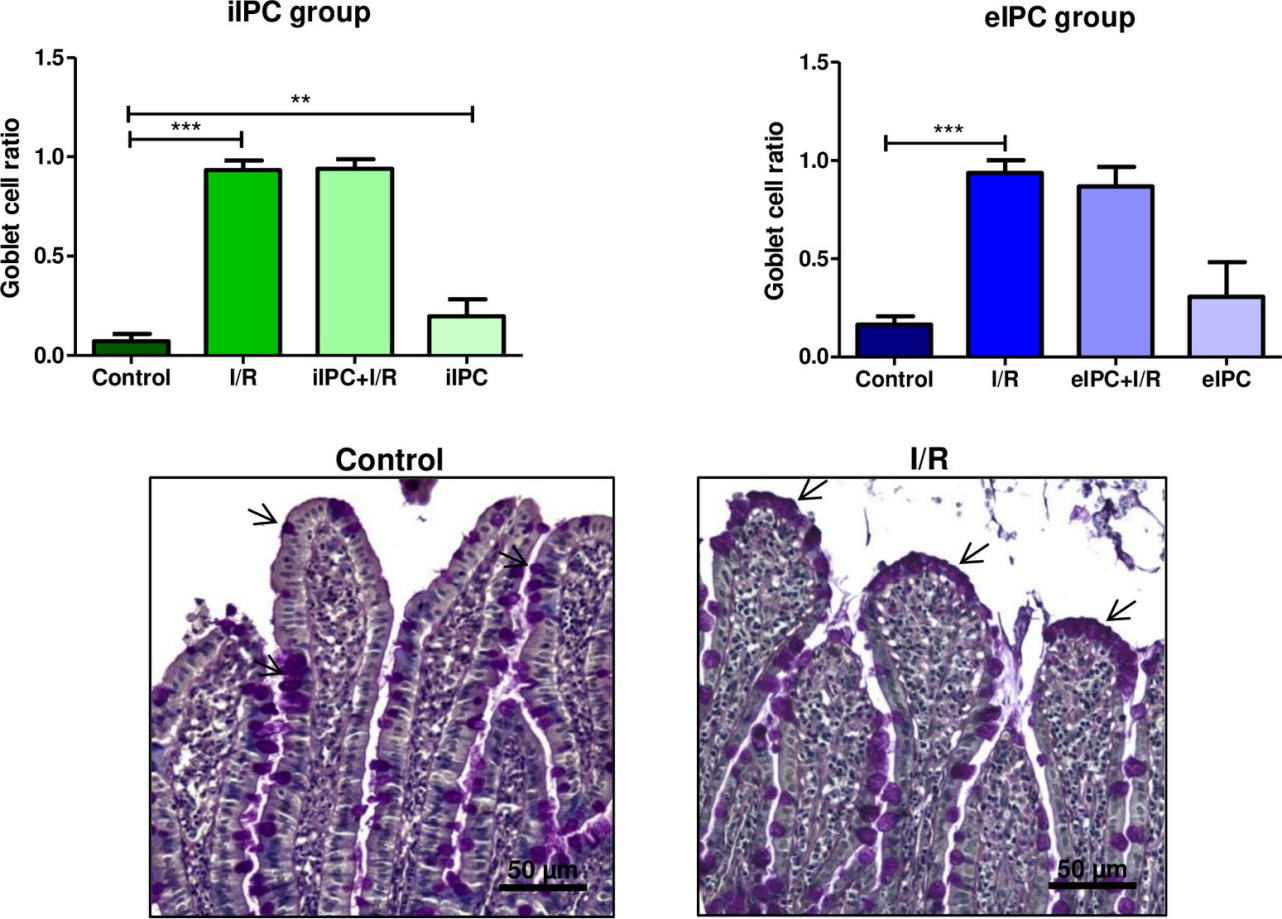
Goblet cell ratio at the end of the experiment in iIPC and eIPC groups. Bars denote the mean +SD. iIPC: Control (N = 7), I/R (N = 6), iIPC+I/R (N = 8), iIPC (N = 7); eIPC: Control (N = 6), I/R (N = 7), eIPC+I/R (N = 8), eIPC (N = 7). **p<0.01; ***p<0.001. Representative histological images of goblet cell (arrows; purple color, PAS staining) containing intestinal villi are shown in the lower panel.

Histological examinations of intestinal tissue samples did not show any effect of I/R on epithelial cell damage or histological stability score. However, a significant change of intestinal epithelium composition was detectable after I/R, showing an increased goblet cell ratio (iIPC: I/R p<0.001 vs. respective control; eIPC: I/R p<0.001 vs. respective control). Interestingly, performing iIPC alone also significantly increased goblet cell ratio (iIPC p<0.01 vs. respective control; Fig 5).

### Effects of I/R and IPC on Caspase-3/7 activity in luminal effluent

**Fig 6:**
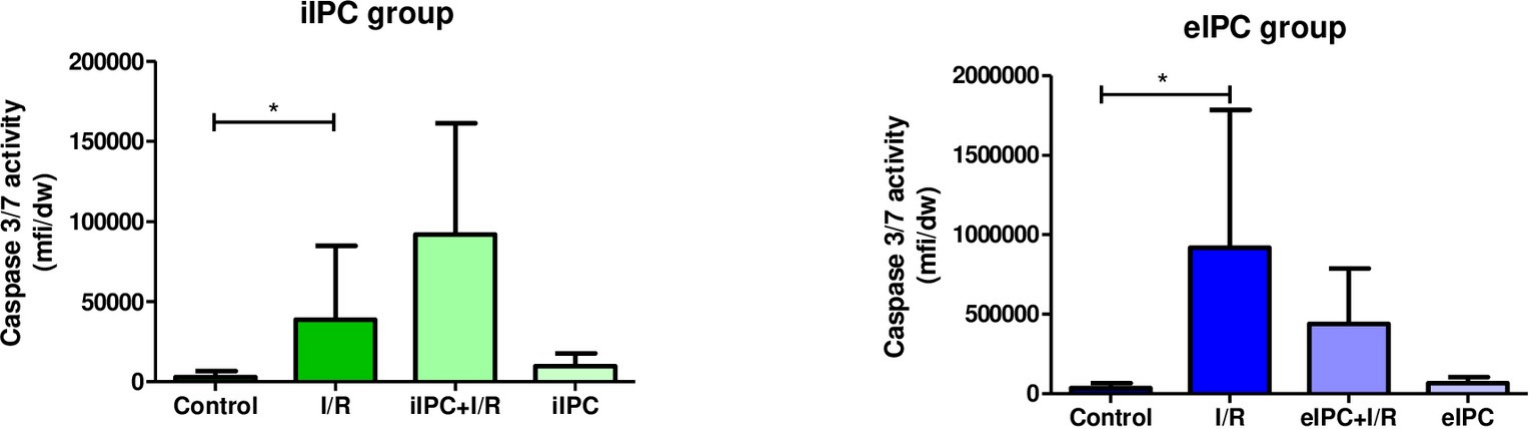
Luminal Caspase 3 and 7 activities at the end of the experiment in iIPC and eIPC groups. Bars denote the mean + SD. iIPC: all (N = 8); eIPC: Control (N = 6), I/R (N = 7), eIPC+I/R (N = 7), eIPC (N = 7). *p<0.05. mfi, mean fluorescent intensity; dw, dry weight of tissue sample.

Caspase-3/7 activity was significantly increased by I/R (iIPC: I/R p<0.05 vs. respective control: eIPC: I/R p<0.05 vs. respective control). However, no influence of iIPC or eIPC on Caspase-3/7 activity was observed (Fig 6).

### Effects of I/R and IPC on vascular perfusion pressure

**Fig 7:**
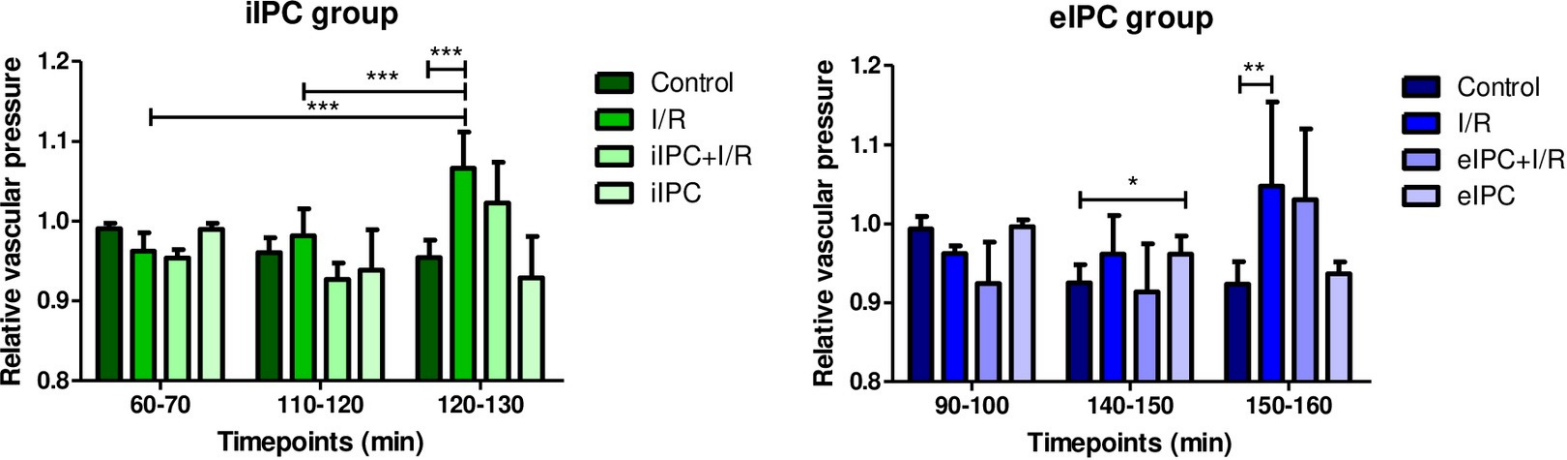
Relative vascular pressure in iIPC and eIPC groups. Bars denote the mean + SD. iIPC: Control (N = 8), I/R (N = 7), iIPC+I/R (N = 9), iIPC (N = 7); eIPC: Control (N = 6), I/R (N = 7), eIPC+I/R (N = 8), eIPC (N = 7). *p<0.05; **p<0.01; ***p<0.001.

Analyses of the relative vascular perfusion pressure revealed a significant increase by I/R during the phase of reoxygenation (iIPC: I/R_t120-130_ p<0.001 vs. respective control; eIPC: I/R_t150-160_ p<0.001 vs. respective control). Interestingly, eIPC alone also resulted in an increase in vascular perfusion pressure (eIPC_t140-150_ p<0.05 vs. respective control; Fig 7).

### Effects of I/R and IPC on venous flow rate

**Fig 8:**
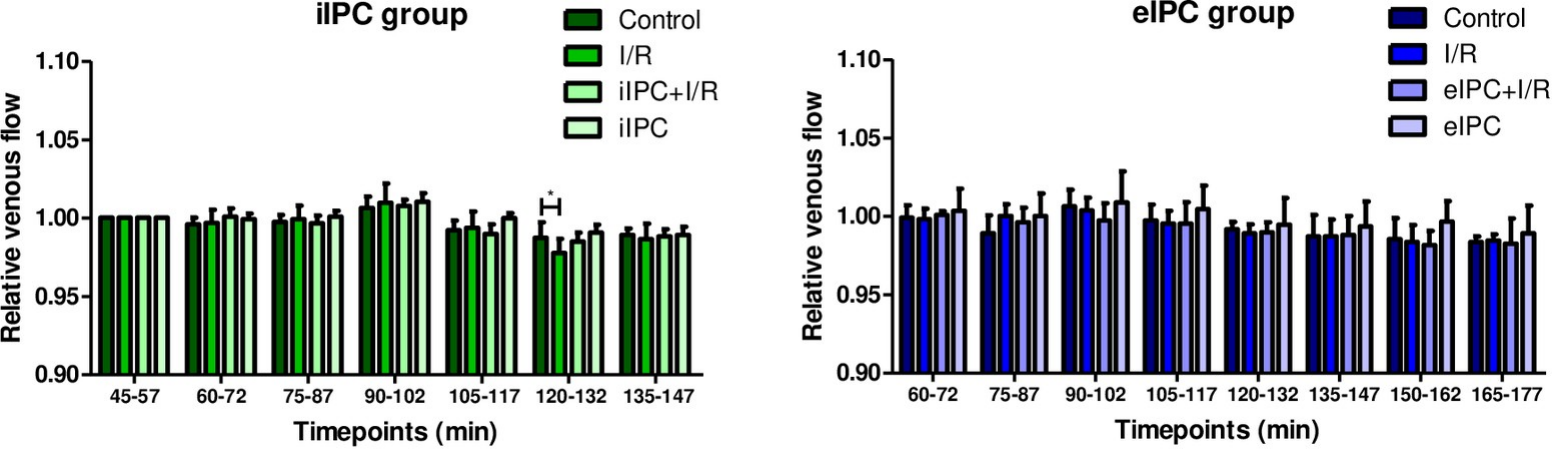
Relative venous flow in iIPC and eIPC groups. Bars denote the mean + SD. iIPC: Control (N = 7), I/R (N = 7), iIPC+I/R (N = 8), iIPC (N = 7); eIPC: Control (N = 6), I/R (N = 6), eIPC+I/R (N = 6), eIPC (N = 5). *p<0.05.

To evaluate fluid shift and barrier integrity between the vascular, interstitial and lymphatic compartment, all relevant effluent flow rates were analyzed. No major influence of I/R was detected. However, I/R reduced the relative venous flow rate temporarily in the early phase of reoxygenation (iIPC: I/R_t120-132_ p<0.05 vs. respective control; Fig 8).

### Effects of I/R and IPC on luminal flow rate

**Fig 9:**
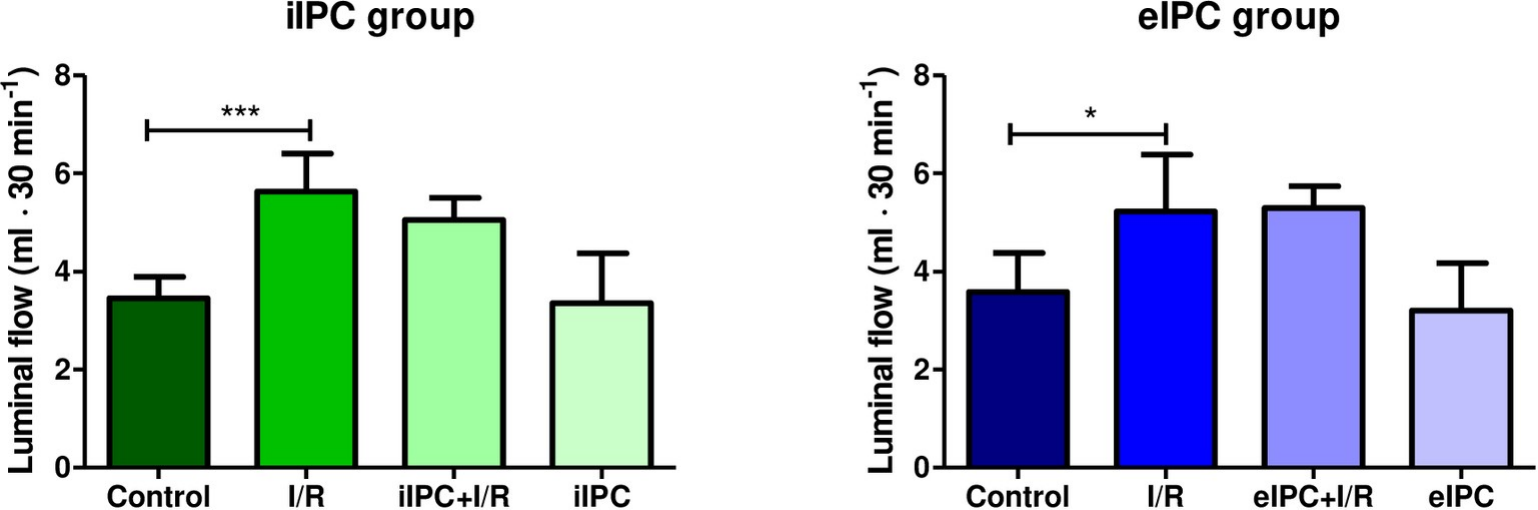
Luminal flow rate at time point t120-150 in iIPC and t150-180 in eIPC groups. Bars denote the mean + SD. iIPC: Control (N = 8), I/R (N = 7), iIPC+I/R (N = 9), iIPC (N = 7); eIPC: Control (N = 6), I/R (N = 7), eIPC+I/R (N = 6), eIPC (N = 7). *p<0.05; ***p<0.001.

Flow rates of all effluents were analyzed to examine fluid homeostasis and barrier integrity. For I/R an increase of the luminal flow rate was observed directly after the hypoxic phase (iIPC: I/R_t120-150_ p<0.001 vs. respective control; eIPC: I/R_t150-180_ p<0.05 vs. respective control), while iIPC and eIPC had no impact (Fig 9).

### Effects of I/R and IPC on luminal FITC-dextran transfer

**Fig 10:**
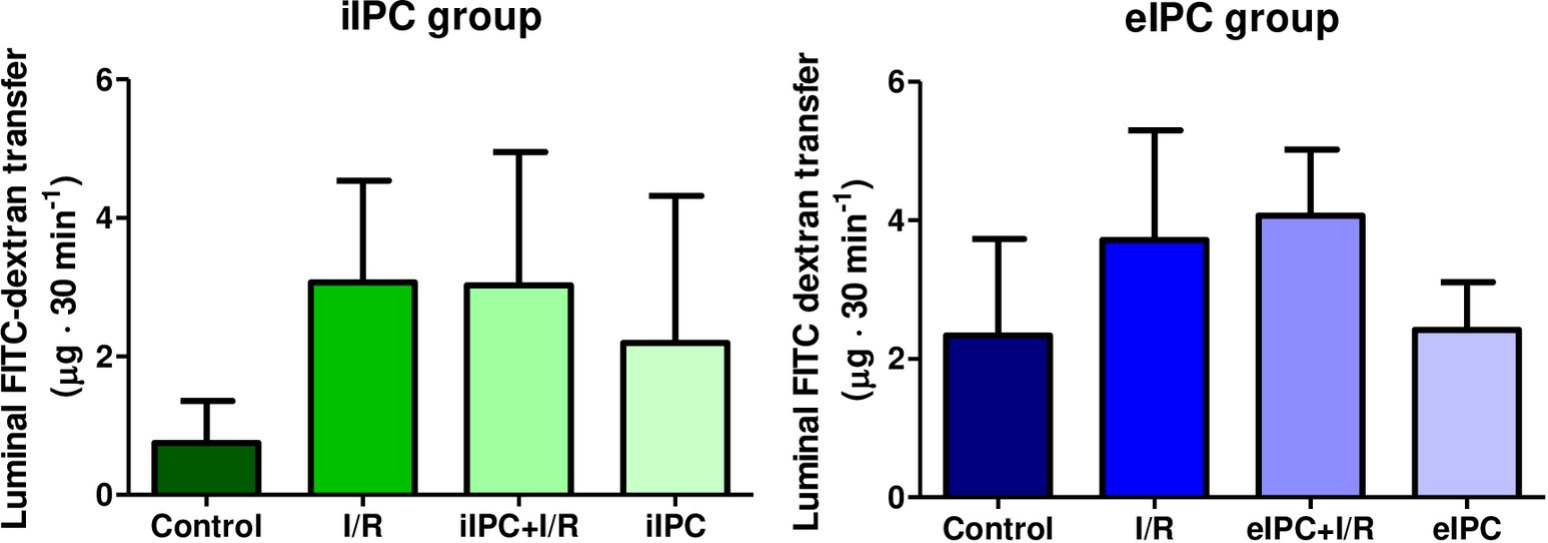
Luminal FITC-dextran transfer at time point t120-150 in iIPC and t150-180 in eIPC groups. Bars denote the mean + SD. iIPC: Control (N = 8), I/R (N = 7), iIPC+I/R (N = 9), iIPC (N = 7); eIPC: Control (N = 6), I/R (N = 7), eIPC+I/R (N = 6), eIPC (N = 7).

To monitor endothelial and epithelial barrier permeability, a known concentration of FITC-dextran was applied to the vascular buffer and FITC-dextran transfer was evaluated in the luminal effluent. I/R increased luminal FITC-dextran in the reoxygenation phase, however, statistical significance was not reached (Fig 10).

### Effects of I/R and IPC on intracellular signaling events

**Fig 11:**
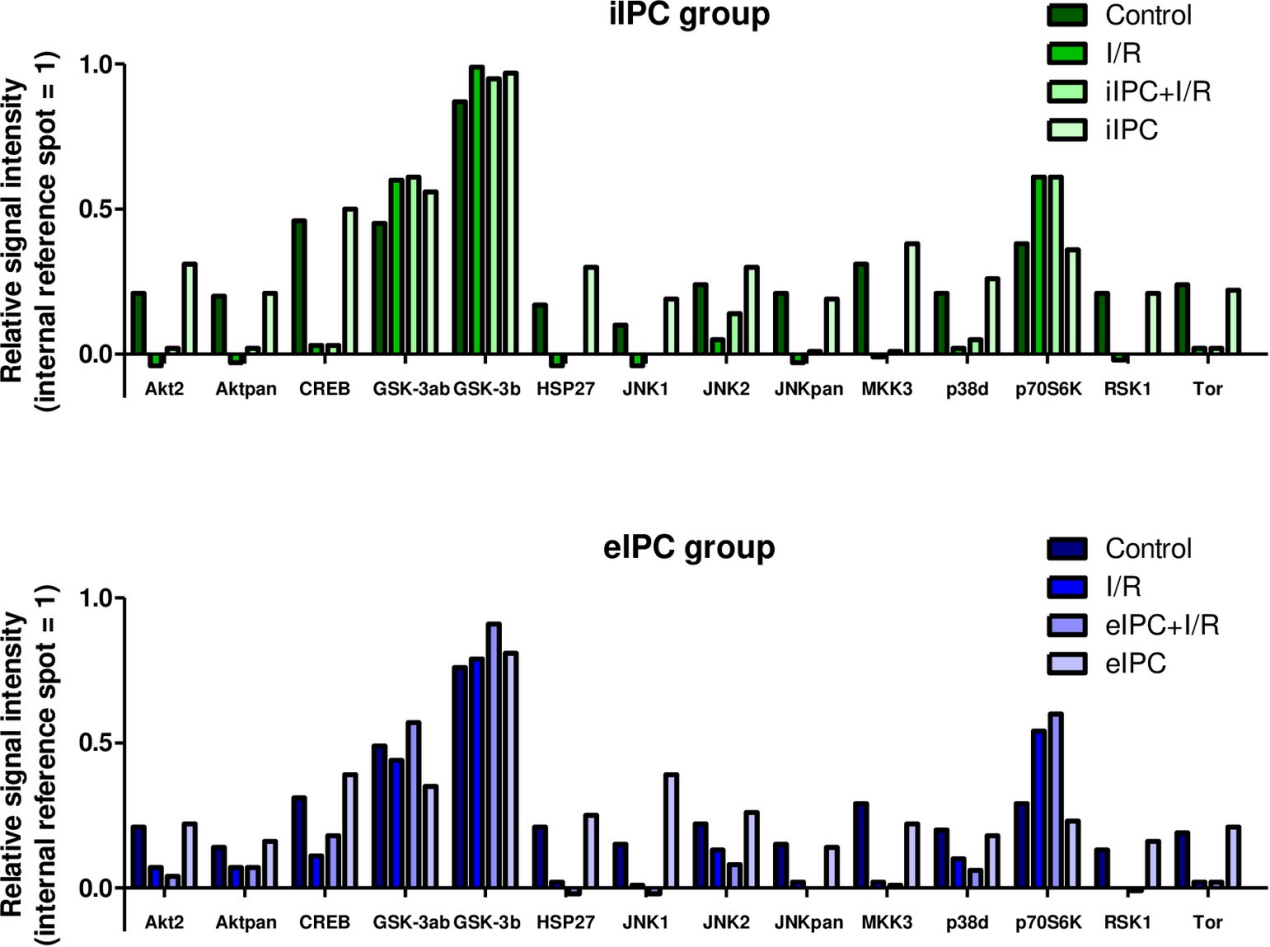
Results of phospho-MAPK arrays from iIPC and eIPC groups.

On cellular level I/R regulated phosphorylation of 14 of the 26 MAP-kinases with the highest regulation for AKT2, AKT pan, CREB, HSP27, JNK1, JNK2, JNKpan, MKK3, p38δ, p70S6 Kinase, RSK1 and Tor (>50% change of phosphorylation vs. control group; Fig 11). Except for p70S6 Kinase I/R reduced the phosphorylation of all of the above mentioned proteins. Compared to the phosphorylation status after I/R, iIPC+I/R increased phosphorylation of JNK2 and p38δ. eIPC+I/R reduced phosphorylation of AKT2 and JNK2 while phosphorylation of CREB and GSK-3α/β was to some extent increased. Interestingly, iIPC and eIPC alone also influenced the phosphorylation of several signaling molecules with the highest values for Akt2, HSP27 and JNK1 (iIPC) and JNK1 (eIPC; Fig 11).

## Discussion

Intestinal I/R-injury and the associated pathomechanisms are of major clinical importance and patients affected by intestinal I/R-injury often experience severe sepsis and multi organ failure with high morbidity [19,20]. Although, the intestine is of high relevance and plays a crucial role in barrier function, nutrient resorption and fluid homeostasis, thorough analyses of physiological and cellular effects of intestinal I/R-injury and the efficiency of potential therapies are still relatively scarce [5,6,10,21,22]. Recently our group investigated the effects and cellular mechanisms of I/R-injury and IPC using human in-vitro [23,24] and rat in-vivo studies [25], demonstrating several protective effects of IPC.

In the actual study we have employed a perfused model of the rat small intestine [13,14] to evaluate physiological and cellular effects of intestinal I/R-injury and to compare the influence of in-vivo IPC (iIPC) with ex-vivo IPC (eIPC), in which blood derived factors and nerval regulations are excluded. We hypothesize that iIPC and eIPC differentially affect physiological as well as cellular events triggered by intestinal I/R-injury.

### Physiological and cellular effects of intestinal I/R-injury

Galactose uptake and lactate-to-pyruvate ratio are markers for intestinal metabolic function and both were significantly influenced by ex-vivo hypoxia/normoxia (reoxygenation) which served as surrogate for in-vivo I/R in our model of the perfused intestine. The lactate-to-pyruvate ratio increased rapidly during hypoxic perfusion and reached normal levels directly after reoxygenation. This was expected and has also been shown by other authors [26,27]. In contrast, galactose absorption decreased significantly by hypoxia and revealed no recovery after reoxygenation. Similar effects on intestinal galactose uptake have been described for a vascular perfusion with a hyper-physiological solution of 3% hydroxyethyl starch (HES) [13,17] and may be explained by dysfunction of the intestinal epithelial sodium/glucose co-transporter 1 due to ATP shortage and associated reduction of the sodium/potassium pump activity [28]. We did not detect alterations in wet/dry ratio and histological edema formation after I/R. This can be explained by the experimental setting with a relatively short reperfusion time. Since in the employed perfused intestine model there is no blood flow but a constant flow of fresh buffer, it is possible that the I/R associated effects in the perfused intestine model are more moderate than in the in-vivo situation and thus longer I/R times may be necessary to induce comparably severe tissue damage. Other I/R-studies with reperfusion times between 1h and 6h described changes in myeloperoxidase levels, mucosal injury as well as other physiological parameters [29,30]. Although, I/R induced histological damage was not observed in our model, a significant increase in the number of intestinal epithelial goblet cells was visible after I/R. This finding may explain the lack of effects of I/R on histo-score and cellular injury: Damaged and shedded enterocytes might be replaced by more robust goblet cells which function as epithelial seals [31,32]. These observations also fit with the I/R associated increase of apoptosis (Caspase-3/7 activity) detected in the luminal effluent where damaged and detached intestinal epithelia cells accumulate. Additional effects of I/R-injury were evident on hemodynamic parameters, especially on vascular perfusion pressure, which was increased during reperfusion, possibly as a result of a massive vasoconstriction. Similar effects were also shown in an ex-vivo perfused dog stomach model with vasodilatation during ischemia and increased vascular resistance during reperfusion [33]. These findings are in accordance with the other hemodynamic parameters evaluated, suggesting a reduced venous flow and increased luminal flow rate during reperfusion representing a fluid loss from the vasculature via the endothelium, interstitial space and intestinal epithelium into the luminal compartment of the intestine. I/R-induced barrier dysfunction in our model is supported by the up to 4-fold increased (but statistically not significant) concentrations of vascularly applied FITC-dextran tracer in the luminal effluents. Regarding subcellular signaling mechanisms, I/R resulted in a decrease in the phosphorylation of almost all of the three major families of mitogen-activated protein kinases (ERK1/2, JNK1-3 and p38α/β/δ/γ) as well as other intracellular proteins such as AKT, GSK-3, p-70S6 Kinase, TOR, and CREB. This is in accordance with the results of other groups that suggested a reduced phosphorylation of AKT by induction of I/R in a rat intestinal model [34] and the enhanced degradation of cAMP response element binding protein (CREB) under hypoxic conditions [35]. However, it should be mentioned that there are also several studies that showed an increased activation of MAPKs and downstream pathways by environmental stressors like LPS, cytokines and also I/R [36–38].

### Physiological and cellular effects of iIPC and eIPC on I/R-injury

The two different IPC strategies (iIPC and eIPC) employed in our ex-vivo perfused rat small intestine model revealed only minor effects on I/R induced alterations in the rat intestine. iIPC significantly increased galactose uptake before I/R but showed no beneficial effects during or after I/R. While other animal studies employed reperfusion periods between 1h and 6h [29,39], our experimental design employing a model of the perfused intestine, allows us only to observe short-term effects of iIPC and eIPC on I/R. This apparent disadvantage of the model is, however, accompanied by many benefits, such as defined and reproducible experimental conditions as well as the advantage of a simultaneous analysis of different cellular and physiological parameters at different time points. Nevertheless, we cannot exclude that a prolonged reperfusion time may reveal additional information especially for parameter with time-delay like Caspase-3/7 activity or galactose uptake. While 1h of ischemia is a widely used experimental time frame in-vivo [40,41], we can also not exclude that the intestinal damage induced by 1h hypoxia/ischemia ex-vivo was already too severe to allow the detection of protective effects of the employed IPC strategies (iIPC and eIPC). Furthermore, ischemic episodes, which are unavoidable during organ preparation and isolation might have to some extent masked potential iIPC or eIPC effects in our model of the perfused intestine.

Despite the rather discouraging effects of iIPC and eIPC on I/R associated physiological parameters, MAPK arrays revealed an increased phosphorylation of JNK2 and p38δ when iIPC was applied prior to I/R. Both molecules are part of the MAPK family and several studies showed JNK and p38 activation by environmental stressors like cytokines and I/R [36–38]. Murayama et al. also demonstrated proapoptotic effects of activated JNK and p38 in the small intestine [36] while Sugden et al. described an I/R related activation of both kinases in the isolated rat heart [38]. These possibly proapoptotic effects may also explain the increased Caspase-3/7 activity found in the luminal effluent. Effects of eIPC on I/R associated physiological and cellular effects were even less pronounced and none of the I/R affected parameters was significantly influenced by eIPC. In addition to the fact that blood derived factors and nerval regulation are both absent in the eIPC group, several of the reasons discussed above also apply to the eIPC experiments and might at least partially explain why in our perfused intestine model eIPC did not modulate effects induced by I/R.

Based on its ex-vivo character, our study has some model related limitations which need to be considered: (i) We cannot exclude the possibility that ligation of the smaller vessels of the intestine before removal of the organ may have caused short ischemic phases in the organ which in turn might mask or interfere with potential iIPC or eIPC effects in our model. (ii) Another limitation of the study is the use of Sevoflurane for anesthetic management. Sevoflurane is considered to have organ-protective properties which could have an influence on the results of the present study. However, it should be noted that other volatile anesthetics as well as propofol have also been ascribed organ-protective properties and to the best of our knowledge, there are no anesthetics available for small animal anesthesia that do not exert organ-protective effects. (iii) We have employed a single pass ex-vivo model lacking recirculating humoral factors, systemic immune cells and accumulation of metabolites. Moreover, our system only allows the observation of short-term effects of I/R-injury and IPC since ex-vivo organ viability is limited. (iv) To ensure a continuous measurement of all parameters, the model is based on a hypoxic perfusion with constant flow rate and low O_2_ partial pressure, while ischemia in-vivo is mostly represented by a low flow or no flow condition. (v) Due to methodological constraints, the length of time the intestine remained in the perfusion chamber differed between the two groups (iIPC group, 150 min; eIPC group, 180 min), and we cannot exclude that this had an influence on the parameters analyzed in the study. (vi) Anesthesia was performed by using Sevoflurane, which exhibits vasodilatory effects. Accordingly, it cannot be excluded that, especially at the beginning of the anesthesia, hypoperfusion may have occurred in some organs/tissues.

## Conclusion

Taken together our results show that although I/R leads to major changes in intestinal physiology, effects of IPC in the acute phase of intestinal I/R-injury are rather limited. It cannot be ruled out that the IPC triggered cellular mechanisms may reveal their protective effects in medium or long-term observations. Therefore, additional animal and clinical studies are needed to assess the significance of IPC for intestinal protection from I/R-injury.

## Supporting information

S1 Fig Representative images of phospho-MAPK arrays from eIPC and eIPC+I/R groups. (TIF)
