# Supplementary figures and images for: Effects of different ischemic preconditioning strategies on physiological and cellular mechanisms of intestinal ischemia/reperfusion injury: Implication from an isolated perfused rat small intestine model
Source: PLoS One. 2021 Sep 3;16(9):e0256957. doi: 10.1371/journal.pone.0256957 (PMC8415612; doi:10.1371/journal.pone.0256957)

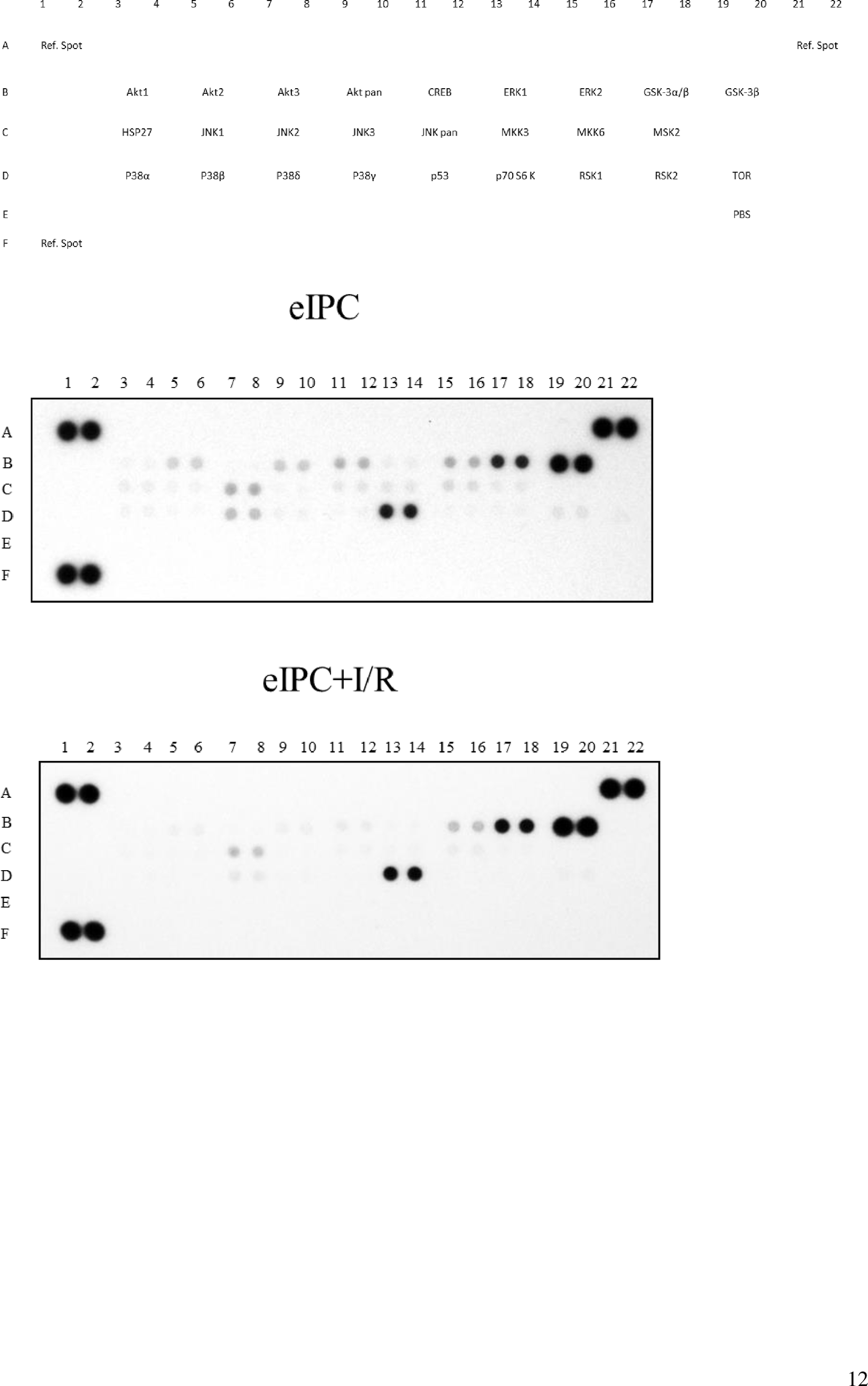

Supplement: S1 Fig — (TIF) [file pone.0256957.s001.tif]
